# Synthesizing porous nanospheres with highly efficient drug loading and sustained release through a thermal-controlled continuous stirred-tank reactor cascade

**DOI:** 10.1039/d5na00897b

**Published:** 2025-12-23

**Authors:** Huiyu Chen, Aniket Pradip Udepurkar, Christian Clasen, Victor Sebastián Cabeza, Simon Kuhn

**Affiliations:** a Department of Chemical Engineering, Process Engineering for Sustainable Systems (ProcESS) KU Leuven, Celestijnenlaan 200F Leuven 3001 Belgium simon.kuhn@kuleuven.be; b Department of Chemical Engineering, Massachusetts Institute of Technology 77 Massachusetts Avenue Cambridge MA 02139 USA; c Department of Chemical Engineering, Soft Matter, Rheology and Technology (SMaRT) KU Leuven, Celestijnenlaan 200J Leuven 3001 Belgium; d Instituto de Nanociencia y Materiales de Aragón (INMA), CSIC-Universidad de Zaragoza Zaragoza 50009 Spain; e Department of Chemical and Environmental Engineering Universidad de Zaragoza Campus Rio Ebro 50018 Zaragoza Spain; f Laboratorio de Microscopías Avanzadas, Universidad de Zaragoza 50018 Zaragoza Spain; g Networking Research Center on Bioengineering, Biomaterials and Nanomedicine (CIBER-BBN) 28029 Madrid Spain

## Abstract

Nanospheres hold great promise for drug delivery but face challenges in achieving both high drug loading and sustained release. Here, we present a novel approach to produce porous cyclosporin A-loaded poly(lactic-*co*-glycolic acid) (PLGA) nanospheres *via* a thermal-controlled continuous stirred-tank reactor (CSTR) cascade, featuring rapid solidification of nanoemulsion droplets. This process traps more drug molecules in the nanosphere core by limiting their diffusion towards the surface and surrounding medium, resulting in a core-loaded structure. The resulting PLGA nanospheres exhibit a high cyclosporin A loading capacity and enable sustained drug release through the hydrolytic degradation of the PLGA matrix. Moreover, the total synthesis time is reduced from several hours to 40 min. The CSTR assisted manufacturing approach offers an efficient route for engineering nanospheres with high drug payloads and improved release kinetics, with broad potential for nanomedicine manufacturing.

## Abstract 2

Advancing drug-loading efficiency and sustained release in PLGA nanospheres through a core-loading strategy, enabled by an engineering-optimized, temperature-controlled CSTR cascade for continuous production. Created with Biorender.com.

## Graphical abstract

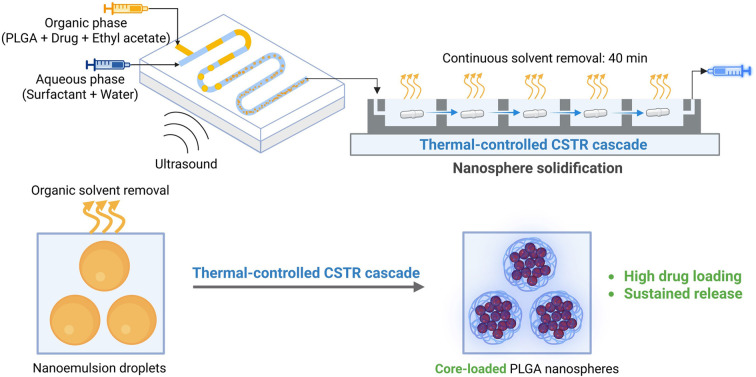

## Introduction

Drug-loaded nanospheres represent a powerful strategy for delivering active pharmaceutical ingredients using inert nanoscale carriers. These nanospheres can protect drug molecules from degradation and promote their transport across biological barriers to reach a target site.^1–4^ The inert materials, such as biodegradable polymers, enable sustained drug release, thus allowing for tailored pharmacokinetic properties and reducing the need for repeated dosing.^5,6^ Moreover, increasing the drug load per nanosphere allows higher doses per injection, improving patient compliance and minimizing excipient-related side effects.^7–9^ For many treatments, achieving a high therapeutic mass fraction is a prerequisite due to strict volume limits on injectable formulations (0.1 mL intradermal, 1 mL subcutaneous, and 1–3 mL intramuscular).^10^ These constraints make the development of nanospheres with high drug content critically important.

Engineering high drug-loaded nanospheres is challenging due to inherent limitations arising from their high surface-to-volume ratio. Drugs positioned on the particle surface are susceptible to loss during the formulation and post-formulation processes.^11–13^ Nanospheres are primarily composed of non-therapeutic scaffold polymers, such as poly(lactic-*co*-glycolic acid) (PLGA). Drug loadings in most reported studies are lower than 4%; some are even substantially below 1%.^11,14–27^ Various strategies have been developed to improve drug loading efficiency through strengthening drug–carrier interactions, such as donor–receptor coordination and covalent conjugation.^28–31^ However, these approaches are limited, as they require both the drug and carrier molecules to have specific structural and chemical properties.

**Fig. 1:**
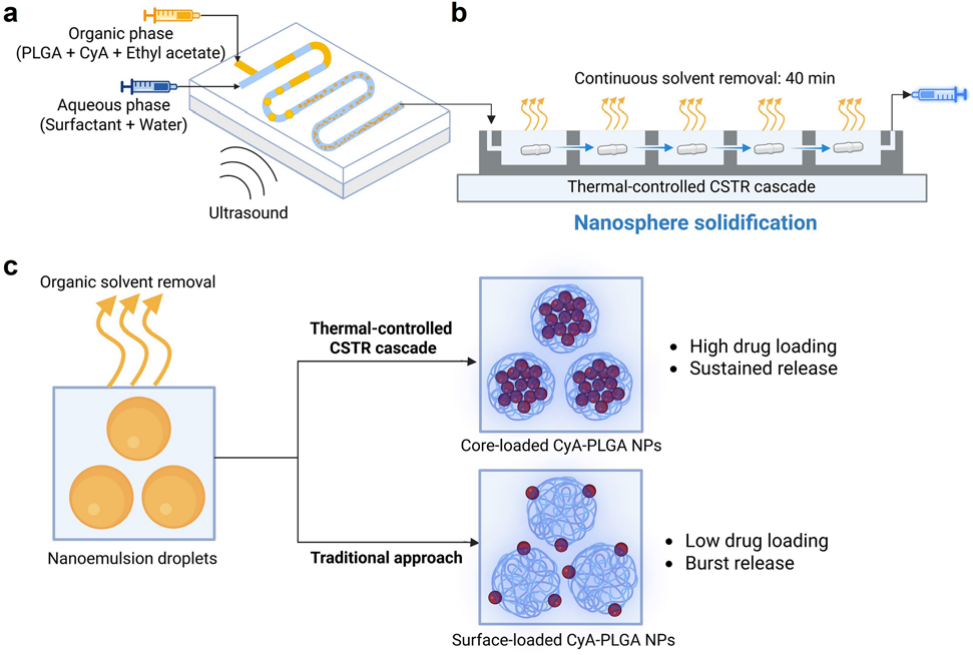
Synthesis process of cyclosporin A-loaded PLGA nanospheres (CyA-PLGA NPs). The setup consists of (a) an ultrasonic microreactor for emulsion generation and (b) a continuous thermal-controlled CSTR cascade for solvent removal and nanosphere solidification. (c) Solvent removal and solidification processes at the droplet/particle level, along with the resulting nanosphere properties, compared to the traditional batch approach. The illustration was created with Biorender.com.

Encapsulating a larger fraction of drug molecules within the nanosphere core, rather than leaving a significant portion on the surface, helps overcome surface-to-volume ratio constraints and enhances overall drug loading. This strategy eliminates the dependence on specific molecular features of the drug and carrier. A core-loaded nanosphere structure not only increases the total drug content but also offers better control over the drug release. Drug release from nanospheres occurs in two phases: an initial burst release caused by the rapid diffusion of surface-located drugs, followed by a more sustained release through the hydrolytic degradation of the PLGA matrix.^32–34^ By increasing the amount of drug loaded in the core, the initial burst release and resulting toxicity risks can be reduced, leading to a more sustained and prolonged release profile. Nanospheres could be formed from nanoemulsion droplets as the organic solvent evaporates (Fig. 1c), leading us to reasonably hypothesize that the solvent removal process would play a critical role in determining the solidification of drug and polymer molecules. By optimizing this process, it may be possible to fabricate core-loaded nanospheres with high drug loading and improved release kinetics.

For continuous nanosphere synthesis, microfluidic nanoprecipitation is used in many studies.^6,35–38^ Although this technique can produce ultra-small nanospheres ranging from 20 to 100 nm, it struggles with a low drug loading capacity (<4%).^14,39–41^ Increasing the particle size can improve drug loading, but it will compromise the ability to cross biological barriers. This hinders particular applications like brain-targeted delivery, which requires particles to be below 100 nm to cross the blood–brain barrier.^42–44^ Furthermore, the small channel sizes required for rapid mixing in microfluidics can lead to issues like low throughput and microchannel clogging.^45–47^

Another commonly used synthesis method is emulsion-solvent evaporation. In 2007, Budhian *et al.*^20^ attempted to increase drug loading by adjusting the pH during solvent evaporation to reduce drug diffusion into the aqueous phase. However, they achieved a maximum drug loading of 2.5%. In 2016, de Solorzano *et al.*^48^ used microchannel emulsification for high-throughput synthesis (∼10 g h^−1^) of cyclosporin A-loaded PLGA nanospheres. However, the resulting particles had a mean size far exceeding 200 nm. Recently, Operti *et al.*^49^ introduced an inline sonicator for continuous emulsification, but the solvent evaporation was performed in a batch mode with dilution and stirring for 1 hour, and the smallest mean particle size achieved was 184.7 nm. To conclude, the synthesis of drug-loaded PLGA nanospheres through emulsion-solvent evaporation has not been fully transitioned to a continuous process. Achieving a mean particle size under 100 nm remains challenging, and the impact of solvent evaporation temperature on drug encapsulation was largely overlooked in previous studies.

In this work, we control the solvent removal process using a continuous stirred-tank reactor (CSTR) cascade (Fig. 1b), which provides thermal regulation, to solidify the drug and polymer molecules. This approach contrasts with previous studies,^48–51^ where solvent removal was performed in batch mode and remained largely unoptimized. This continuous manufacturing strategy eliminates batch-to-batch variations and provides a well-defined scale-up pathway,^52,53^ addressing two major challenges in the industrialization of nanomedicines.^54–58^ To validate our approach, cyclosporin A (CyA) is selected as a model peptide drug due to its poor water solubility and limited bioavailability (class II from the Biopharmaceutical Classification System, BCS). The solvent removal step is systematically optimized, and its impact on particle size distribution, morphology, and drug loading efficiency of cyclosporin A-loaded PLGA nanospheres (CyA-PLGA NPs) is assessed. Furthermore, *in vitro* drug release studies are conducted to evaluate the sustained release profile. Process efficiency and residual solvent content are also analysed to ensure the robustness and scalability of the developed method.

## Results and discussion

### Synthesis and characterization of CyA-PLGA NPs

CyA-PLGA NPs were synthesized using the emulsion–solvent evaporation technique. In the first step, an automated ultrasonic microreactor was employed to generate nanoemulsion droplets. The organic phase, consisting of PLGA and cyclosporin A dissolved in ethyl acetate, and the aqueous phase, consisting of the surfactant Poloxamer 407 in Milli-Q water, were introduced into the microreactor. Ultrasonic energy was applied *via* a piezoelectric transducer plate attached to the bottom of the reactor to facilitate emulsification (Fig. 1a). Following droplet formation, the nanoemulsion was fed into a CSTR cascade for continuous thermal-controlled solvent removal (Fig. 1b). The outlet of the CSTR cascade was connected to a syringe pump, which continuously withdrew the solidified CyA-PLGA NP suspension. The temperature of the CSTR cascade was regulated using a circulating water jacket located beneath the wells (Fig. 3a). The entire process was automated and conducted at a constant flow rate of 250 µL min^−1^, with an overall residence time of ∼40 min. For comparison, a traditional batch method was carried out by collecting the nanoemulsion from the microreactor outlet into a glass vial (Fig. 3b), followed by magnetic stirring for at least 4 hours at room temperature to allow for solvent evaporation.

**Fig. 2:**
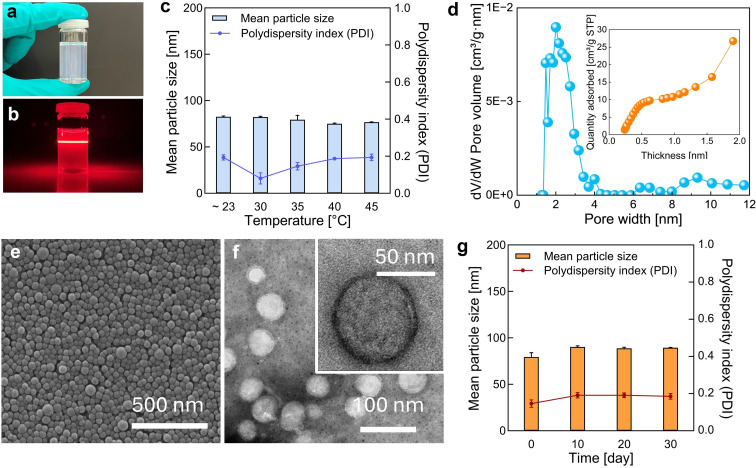
Characterization of CyA-PLGA NPs. (a) Visual appearance of the CyA-PLGA NP suspension. (b) Tyndall effect observed in the suspension using a laser pointer. (c) Mean particle size and polydispersity index (PDI) measured by dynamic light scattering (DLS). (d) Porosity distribution determined by density functional theory (DFT) analysis and *t*-plot analysis of CyA-PLGA NPs based on Brunauer–Emmett–Teller (BET) isotherms, measured at standard temperature and pressure (STP, 273 K and 101.325 kPa) with N_2_. Representative (e) scanning electron microscopy (SEM) image, (f) transmission electron microscopy (TEM) images, and (g) stability profile over 30 days (storage 2–8 °C) of CyA-PLGA NPs prepared *via* the thermal-controlled CSTR cascade at 35 °C. Data are presented as mean ± SD (*n* = 3).

For effective drug delivery, nanospheres smaller than 200 nm with a polydispersity index (PDI) below 0.2 are desired,^59,60^ while brain-targeted therapies typically demand even smaller sizes—under 100 nm.^42,44,61^ To meet these criteria, the emulsification step in the ultrasonic microreactor was systematically optimized (Table S2) before integrating the thermal-controlled CSTR cascade. After implementing the CSTR cascade for continuous solvent removal, the process temperature was varied from room temperature (∼23 °C) to 45 °C. Across this temperature range, the resulting CyA-PLGA NPs consistently maintained a mean particle size below 82 nm and a PDI below 0.2, fulfilling the size distribution requirements for biomedical applications. The pore size distribution results from the Brunauer–Emmett–Teller (BET) analysis (Fig. 2d) indicate the presence of micropores around 1.5 nm and mesopores in the 2–3 nm range. Larger pores (>5 nm) are likely due to interstitial voids between the nanospheres. The coexistence of micropores and mesopores is further confirmed by the *t*-plot curve, which exhibits a characteristic profile typical of micro–mesoporous materials.^62^ SEM and TEM images revealed a smooth, spherical morphology with a uniform size distribution. No significant morphological differences were observed between nanoparticles synthesized at ∼23 °C (Fig. S10) and at 35 °C (Fig. 2e and f), indicating that the mild thermal treatment does not compromise particle integrity. Additionally, the CyA-PLGA NPs demonstrated good storage stability, retaining their size distribution for at least 30 days when stored at 2–8 °C (Fig. 2g).

### Thermal-controlled CSTR cascade for enhanced drug loading

**Fig. 3:**
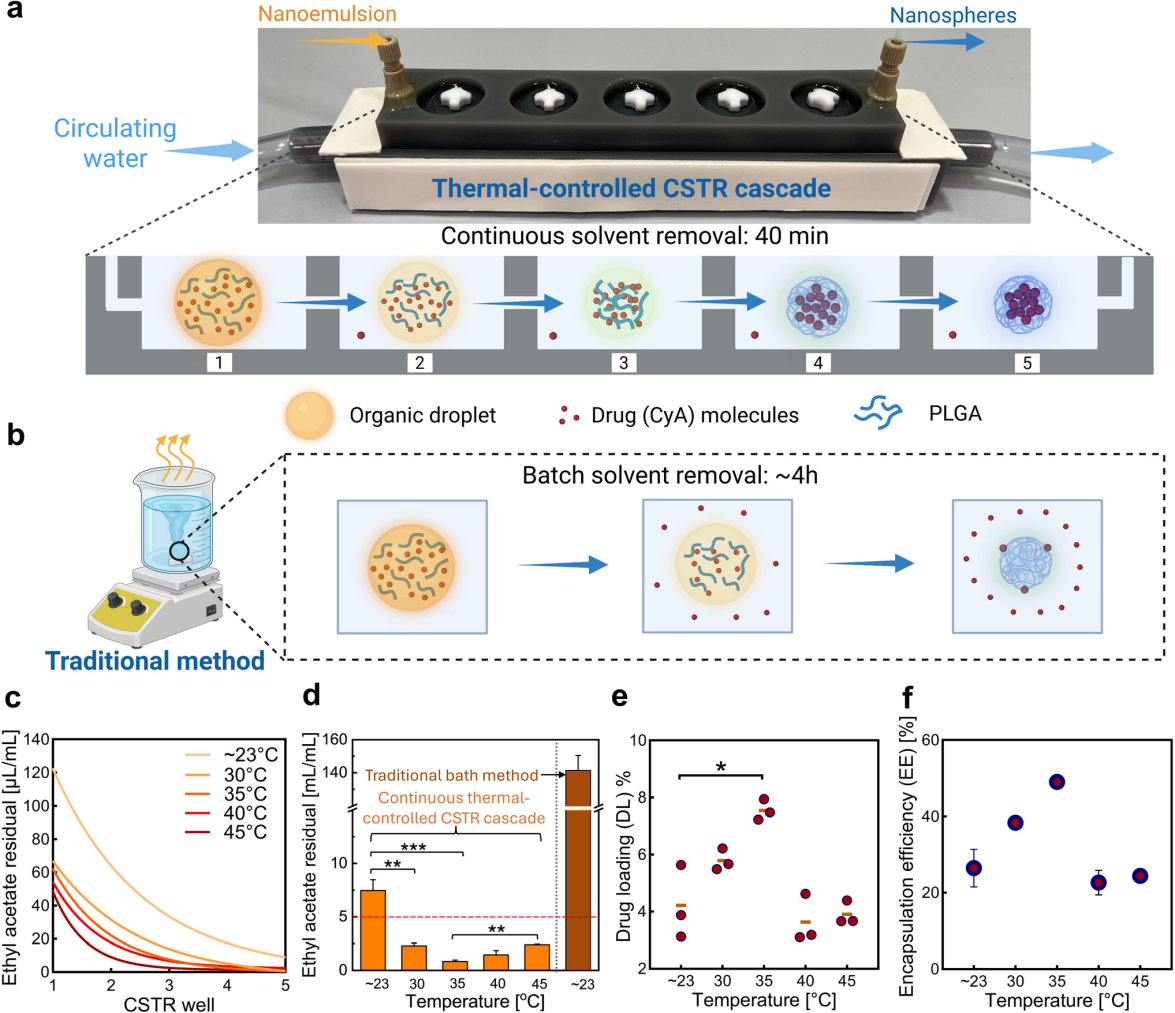
Optimization of the thermal-controlled CSTR cascade and the resulting enhancement in drug loading efficiency. Schematic comparison of the organic solvent removal and nanoparticle formation process using (a) thermal-controlled CSTR cascade and (b) traditional batch method. (c) Residual ethyl acetate concentrations measured in each CSTR well (1–5) during the solvent removal process. (d) Final residual solvent content in the nanoparticle suspension. (e) Drug loading (DL) and (f) encapsulation efficiency (EE) of the resulting CyA-PLGA NPs obtained at varying CSTR cascade temperatures (∼23 °C to 45 °C). In graphic (d), the red dashed line indicates the maximum allowed residual level of ethyl acetate according to EMA guidelines (5 µL mL^−1^). Data are shown as mean ± SD, *n* = 3. Statistical significance is indicated as *p < 0.05, **p < 0.01, and ***p < 0.001. Only relevant and significant statistical comparisons are highlighted.

The solvent removal time was significantly reduced from over 4 hours (ref. 63) to just 40 min by replacing the conventional batch method (Fig. 3b) with a continuous CSTR cascade (Fig. 3a), which doubled the surface-to-volume ratio (Table S1). At a fixed duration of 40 min and room temperature (∼23 °C), the residual ethyl acetate concentration decreased from 141.3 µL mL^−1^ (batch) to 8.0 µL mL^−1^ (CSTR), demonstrating improved efficiency. However, to meet EMA regulations for Class 3 solvents,^64^ which require residual levels below 5 µL mL^−1^ (5000 ppm), faster solvent removal is necessary. This was addressed by introducing thermal control at elevated temperatures.

The residual ethyl acetate content in each CSTR well during solvent removal (Fig. 3c) and in the final nanosphere suspension (Fig. 3d), along with the drug loading (DL, Fig. 3e) and encapsulation efficiency (EE, Fig. 3f) of the resulting CyA-PLGA NPs, was quantified by HPLC. The results showed that at 30 °C and 35 °C the final residual ethyl acetate was significantly reduced to 2.3 ± 0.3 µL mL^−1^ and 0.9 ± 0.3 µL mL^−1^, respectively, both well below the 5 µL mL^−1^ threshold. Interestingly, a slight increase in residual solvent was observed at 40 °C (1.5 ± 0.4 µL mL^−1^) and 45 °C (2.4 ± 0.1 µL mL^−1^). The drug loading increased from 4.2 ± 1.3% at ∼23 °C to a peak value of 7.6 ± 0.4% at 35 °C, but then declined to 3.6 ± 0.7% and 3.9 ± 0.3% at 40 °C and 45 °C, respectively, even below the value at room temperature (∼23 °C). A similar trend was observed for the encapsulation efficiency, which rose from 26.5 ± 8.5% to 49.0 ± 2.5% at 35 °C and then decreased to 22.7 ± 4.6% and 24.4 ± 2.2% at the higher temperatures.

The initial reduction in residual ethyl acetate at moderately elevated temperatures is attributed to an accelerated solvent removal rate, as further supported by measurements from individual CSTR wells (1–5) during the process (Fig. 3c). The observed improvement in drug loading efficiency results from more efficient solvent removal achieved through the thermal-controlled CSTR cascade. During this process, the organic solvent must first diffuse from the emulsion droplets into the surrounding aqueous phase before it can evaporate.^65,66^ Katou *et al.*^67^ proposed a mathematical model to describe the kinetics of this solvent transfer from oil droplets to the aqueous phase:1
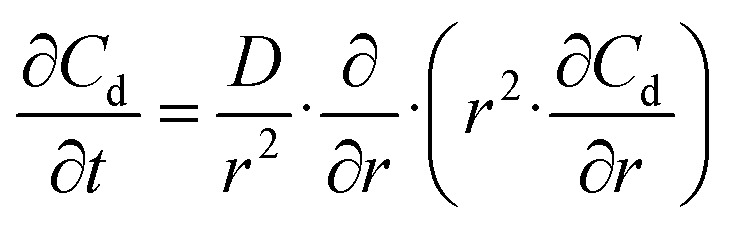
where *C*_d_ stands for the solvent concentration, *t* stands for time, *r* stands for radial position, and *D* stands for the solvent diffusion coefficient. According to the Wilke–Chang equation, the solvent diffusion coefficient *D* is given by2
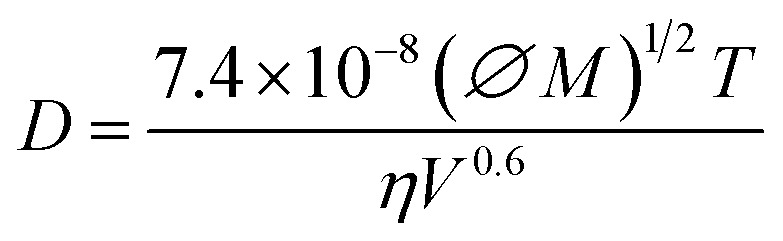
where *∅* is the association parameter, *M* is the molecular mass of the solvent, *T* is the temperature, *η* is the viscosity, and *V* is the molecular volume. The diffusion coefficient is proportional to the temperature, meaning that at higher temperatures, solvent removal occurs more rapidly. This accelerates droplet solidification and limits the time available for drug diffusion into the external aqueous phase. As a result, a larger amount of the drug is retained within the polymeric matrix of the nanospheres, significantly enhancing drug loading.

However, at further elevated temperatures of 40 °C and 45 °C—surpassing the glass transition temperature 
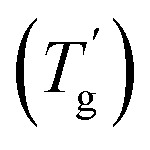
 of PLGA (Fig. S11)—the polymer does not solidify into a rigid, glassy state but instead transitions into a softer, rubbery phase.^68–70^ In this rubbery state, stronger polymer–solvent interactions hinder solvent diffusion and evaporation, resulting in higher residual ethyl acetate levels despite the elevated temperature. Moreover, this rubbery state leads to two major effects: (i) the diffusion of the organic solvent slows down considerably, giving the drug more time to diffuse out of the polymeric matrix and escape into the aqueous phase, reducing drug retention; (ii) in some cases, the polymer–drug mixture fails to solidify into nanoparticles before reaching the liquid surface during evaporation. Instead, it forms a polymeric film on the liquid surface in the CSTR (as observed during experiments), leading to a loss of the drug material and reduced nanoparticle formation. Consequently, the drug loading efficiency at 40 °C and 45 °C declined, even falling below that achieved at room temperature (∼23 °C).

Therefore, 35 °C was determined to be the optimal solvent removal temperature in the CSTR cascade, as it enables ideal particle size distribution, maximizes the drug loading efficiency, and minimizes the residual ethyl acetate content, while preventing the detrimental effects of PLGA transitioning into its rubbery state above 
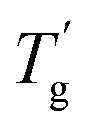
.

To confirm that these improvements were due to the nature of the thermal-controlled CSTR cascade nanotechnology, rather than temperature alone, a batch solvent removal was also performed at 35 °C for comparison (Table S4). When the batch process was run for the same duration as that of the CSTR cascade (40 min), the drug loading efficiency was extremely low at 0.8 ± 0.1%, indicating that almost no drug was encapsulated. As previously discussed, this is attributed to the poor solvent removal efficiency of the batch method, which left a high residual ethyl acetate concentration (91.9 ± 1.6 µL mL^−1^). With the aqueous phase nearing saturation, the diffusion of ethyl acetate from the droplets was restricted, significantly slowing solidification. This prolonged diffusion window allowed drug molecules to escape into the aqueous phase before being entrapped. Even after extending the batch evaporation to 4 hours, the drug loading efficiency only modestly increased to 2.5 ± 0.9%, still far below the value achieved with the CSTR cascade. These results confirm that the significant improvement in drug loading was primarily due to the efficient solvent removal enabled by the thermal-controlled CSTR cascade.

### *In vitro* drug release

**Fig. 4:**
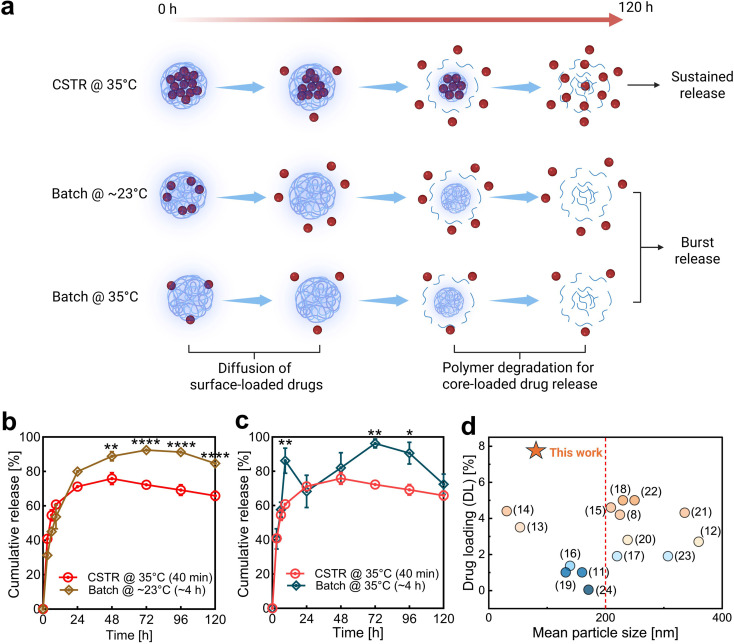
*In vitro* drug release demonstrating the advantage of the core-loaded nanoparticle structure. (a) Schematic illustration of the two-phase drug release mechanism from PLGA nanoparticles. Sustained and burst release profiles resulting from core-loaded and surface-loaded drug distributions, respectively. (b and c) *In vitro* drug release data of CyA-PLGA NPs prepared *via* the thermal-controlled CSTR cascade at 35 °C (CSTR @ 35 °C), compared to those prepared using the traditional batch method at room temperature (Batch @ ∼23 °C) and at 35 °C (Batch @ 35 °C). Data are presented as mean ± SD (*n* = 3). Statistical significance is indicated as follows: *P < 0.05; **P < 0.01; ***P < 0.001; ****P < 0.0001. (d) Positioning our core-loaded CyA-PLGA NPs within the landscape of benchmark studies,^11,14–27^ highlighting their exceptional ability to simultaneously achieve both a high drug loading efficiency and a small particle size (two critical and often mutually exclusive parameters in nanoparticle synthesis). The red dashed line in panel (d) marks the <200 nm threshold typically considered suitable for drug delivery applications.

The release of a poorly water-soluble drug from PLGA nanospheres typically happens in two phases (Fig. 4a). The first phase is a rapid initial burst release, driven by the diffusion of the drug located near the particle surface. The second phase is more sustained and is attributed to the hydrolysis of the PLGA and erosion of the polymeric matrix, which releases the drug encapsulated in the nanosphere core.^32–34^ If the enhanced solvent removal rate indeed results in increased drug encapsulation in the nanosphere core, CyA-PLGA NPs synthesized using the thermal-controlled CSTR cascade at 35 °C should exhibit less of an initial burst release and more drug release during the second sustained phase.

To provide a meaningful comparison, we selected the batch solvent evaporation at ∼23 °C (Batch @ ∼23 °C) to represent the slowest evaporation rate. The drug release profile over 120 h, depicted in Fig. 4b, confirms the hypothesis. During the first 24 hours of the initial release phase, there was no statistically significant difference between the two samples. However, by the end of the burst release phase (72 h), 93% of the drug encapsulated in the Batch @ ∼23 °C nanospheres was released, indicating that a major fraction of the CyA molecules was located close to the surface. In contrast, the CSTR @ 35 °C nanospheres only released 76% of their drug content during the initial burst phase (48 h), leaving 24% encapsulated within the nanosphere core for a slower, sustained release. The cumulative drug release gradually declined in the second phase, as the drug continued to be released, but at a slower rate than its degradation, making the net change appear negative. This trend aligns with the CyA stability profile observed in our degradation test (Fig. S9) and is consistent with findings reported in other studies.^71,72^

Next, we conducted an additional *in vitro* drug release study on CyA-PLGA NPs prepared *via* batch solvent evaporation at 35 °C (Batch @ 35 °C). The aim was to assess whether the core-loaded drug distribution observed in nanospheres produced by the CSTR cascade resulted primarily from the high solvent removal efficiency of the thermal-controlled CSTR cascade process, or if similar characteristics could be achieved by applying the same elevated temperature in a conventional batch process. As shown in Fig. 4c, the Batch @ 35 °C nanospheres exhibited a sharp burst release, with 86% of the encapsulated drug released within the very first 9 h. This release was even more abrupt than that of the Batch @ ∼23 °C nanospheres, suggesting that the drug was located even closer to the particle surface. By 24 hours, the CyA concentration in the release medium had already begun to decline due to degradation, and the drop was more pronounced because there was little to no sustained release between 9 and 24 hours to replenish the drug. In the end, the sustained release phase began after 48 h, corresponding to the release of the remaining 14% of the drug from the nanosphere core.

Based on the drug release results, we can conclude that the Batch @ 35 °C nanospheres contained a larger fraction of the drug encapsulated in the core (14%) compared to the Batch @ ∼23 °C nanospheres (7%), but still less than the CSTR @ 35 °C nanospheres (24%). This suggests that the elevated temperature enhanced solvent diffusion from the droplets into the aqueous phase, allowing more drug to be retained in the nanoparticle core. However, the batch process is limited by a low surface-to-volume ratio, making the evaporation of solvent from the aqueous phase into air the rate-limiting step. This inefficiency resulted in a high residual solvent concentration in the aqueous phase, which in turn suppressed further diffusion of organic solvent from the droplets. Consequently, solvent accumulated at the droplet/nanoparticle interface, providing more time for drug molecules to diffuse into the aqueous phase and be lost, leading to lower drug loading efficiency (2.5 ± 0.9%) compared to the CSTR @ 35 °C (7.6 ± 0.4%). Moreover, this solvent accumulation forces a larger portion of the drug toward the sphere surface, as evidenced by a rapid burst release of 86% of the drug within the first 9 h, in contrast to the slower burst release observed in Batch @ ∼23 °C nanospheres (93% released over 48 h). Therefore, the improvement was not solely due to the elevated temperature, but was primarily driven by the high solvent removal efficiency achieved through the thermal-controlled CSTR cascade.

## Conclusions

In summary, we have developed a thermal-controlled CSTR cascade nanotechnology for the synthesis of CyA-PLGA NPs, achieving a significantly improved drug loading efficiency and a more sustained release profile. The formation of nanospheres with high drug loading is a result of a larger fraction of drug molecules solidifying at the particle core, realized by a more efficient solvent removal in the CSTR cascade at 35 °C. The achieved drug loading is, to our knowledge, the highest reported for sub-100 nm CyA-PLGA NPs, significantly exceeding the previously reported maximum of 4.6%^11,14–27^ (Fig. 4d). The core-loaded structure not only provides a higher drug payload, but also enables gradual release of drug molecules. The sustained release from high drug-loaded nanospheres may contribute to an enhanced therapeutic efficacy and reduced adverse effects for drug delivery applications. Additionally, the robustness and scalability of the thermal-controlled CSTR cascade provide a promising pathway for clinical translation, aligning with FDA guidelines for modern pharmaceutical manufacturing.^73^

## Author contributions

Conceptualization: H. C., A. P. U. and V. S. C. Methodology: H. C., A. P. U. and V. S. C. Experiments: H. C., A. P. U., and V. S. C. Writing – original draft: H. C. and A. P. U. Writing – review and editing: C. C., V. S. C., and S. K. Data curation: H. C. and A. P. U. Supervision: C. C., V. S. C., and S. K. Funding acquisition: S. K.

## Conflicts of interest

There are no conflicts to declare.

## Supplementary Material

NA-OLF-D5NA00897B-s001

## Data Availability

Supplementary information (SI): materials and methods, supplementary results of nanosphere synthesis, images, degradation studies, and numerical data corresponding to the plotted figures. See DOI: https://doi.org/10.1039/d5na00897b.
