# Supplementary material for: Synthesizing porous nanospheres with highly efficient drug loading and sustained release through a thermal-controlled continuous stirred-tank reactor cascade
Source: Nanoscale Adv. 2025 Dec 23;8(4):1281–90. doi: 10.1039/d5na00897b (PMC12802578; doi:10.1039/d5na00897b)
Supplement: NA-008-D5NA00897B-s001 [file NA-008-D5NA00897B-s001.pdf]

Supplementary Information (SI) for Nanoscale Advances.
This journal is © The Royal Society of Chemistry 2026

Supplementary Information

Synthesizing Porous Nanospheres with Highly Efficient Drug Loading and Sustained Release through a Thermal-Controlled Continuous Stirred-Tank Reactor Cascade

Huiyu Chen[a, ∇], Aniket Pradip Udepurkar[a,b, ∇], Christian Clasen[c], Victor Sebastián Cabeza[d,e,f,g], Simon Kuhn[a]*

[a] Department of Chemical Engineering, Process Engineering for Sustainable Systems (ProcESS), KU Leuven, Celestijnenlaan 200F, Leuven 3001, Belgium

[b] Department of Chemical Engineering, Massachusetts Institute of Technology, 77 Massachusetts Avenue, Cambridge, MA 02139, USA

[c] Department of Chemical Engineering, Soft Matter, Rheology and Technology (SMaRT), KU Leuven, Celestijnenlaan 200J, Leuven 3001, Belgium

[d] Instituto de Nanociencia y Materiales de Aragón (INMA), CSIC-Universidad de Zaragoza, Zaragoza 50009, Spain

[e] Department of Chemical and Environmental Engineering Universidad de Zaragoza Campus Rio Ebro, 50018 Zaragoza, Spain

[f] Laboratorio de Microscopías Avanzadas, Universidad de Zaragoza, 50018 Zaragoza, Spain

[g] Networking Research Center on Bioengineering, Biomaterials and Nanomedicine (CIBER-BBN), 28029 Madrid, Spain

* Corresponding author. E-mail address: simon.kuhn@kuleuven.be (S. Kuhn)

[∇] These authors contributed equally to this work.

EXPERIMENTAL SECTION

Materials. PLGA (Resomer® RG 504 H, acid terminated, L:G 50:50, MW 38,000-54,000 Da), surfactant Poloxamer 407 (purified, non-ionic), ethyl acetate ($\geqslant$ 99.5%), and Cyclosporin A was purchased from Sigma-Aldrich. Acetonitrile (HPLC grade) and methanol (anhydrous, 99.8 %) were obtained from VWR, Belgium. Acetonitrile (HPLC grade) and Milli-Q water were employed for the HPLC measurements. Milli-Q water was utilized for all the experiments.

CyA-PLGA NP synthesis: batch solvent removal. The ultrasonic microreactor set-up consists of a borosilicate glass microreactor (fabricated by Little Things Factory GmbH, microchannel cross-section 1.2 × 1.2 mm^2, length 700 mm, reactor volume 1 mL), a piezoelectric plate transducer (C-205, Fuji Ceramics, 80 × 40 × 4 mm^3) glued to the microreactor's bottom, and a Peltier cooling element (RS Components). The plate transducer was bonded to the microreactor using epoxy glue (EPO-TEK® 301, Epotek). Ultrasound was actuated by connecting the transducer to a signal generator (33500B, Keysight) and a power amplifier (RF1040L, 400 W, E&I). The system was operated at a resonance frequency of 550 kHz with adjustable ultrasound power. The Peltier cooling element was controlled by a DC power supply (Velleman) to regulate the outlet temperature at 30°C.

CyA-PLGA NPs were synthesized using the emulsion-solvent evaporation technique in this ultrasonic microreactor. The organic phase, consisting of PLGA and CyA dissolved in ethyl acetate, and the aqueous phase, consisting of surfactant Poloxamer 407 (5 mg/mL) in Milli-Q water, were introduced into the microreactor using syringe pumps (Fusion 200, KR Analytical) via PFA tubing. The total flow rate was set to 250 μL/min, resulting in a residence time of 4 min for all experiments. The organic phase flow rate was 50 μL/min, while the aqueous phase flow rate was 200 μL/min. Ultrasound was applied once segmented flow was established within the microchannel. The oil-in-water emulsion sample was collected in a glass vial and stirred

gently (400 rpm) overnight on a stirring plate (MIXdrive 15, 2MAG) to evaporate the organic solvent, allowing the emulsion droplets to form a suspension of drug-loaded PLGA nanospheres. Surfactants and unencapsulated drug were removed by centrifugation at 10,000 g for 30 min, repeated 5 times, to obtain a purified nanosphere suspension.

CyA-PLGA NP synthesis: thermal-controlled CSTR cascade. For the continuous synthesis of CyA-PLGA NPs, the ultrasonic microreactor used for emulsification was combined with a 3D-printed CSTR cascade for continuous solvent evaporation. Temperature control for the CSTR cascade was achieved by placing it on a glass jacket connected to a circulating water bath. Different solvent evaporation temperatures were investigated. At the bottom, a magnetic stirring plate (VWR, VHP-C7, 180 x 180 mm^2) was set at 250 rpm ensuring uniform mixing via magnetic stirring bars placed in each CSTR well. The CSTR wells were linked by a square channel (5 × 2 × 2 mm^3), allowing continuous flow between them. A design sketch of the CSTR

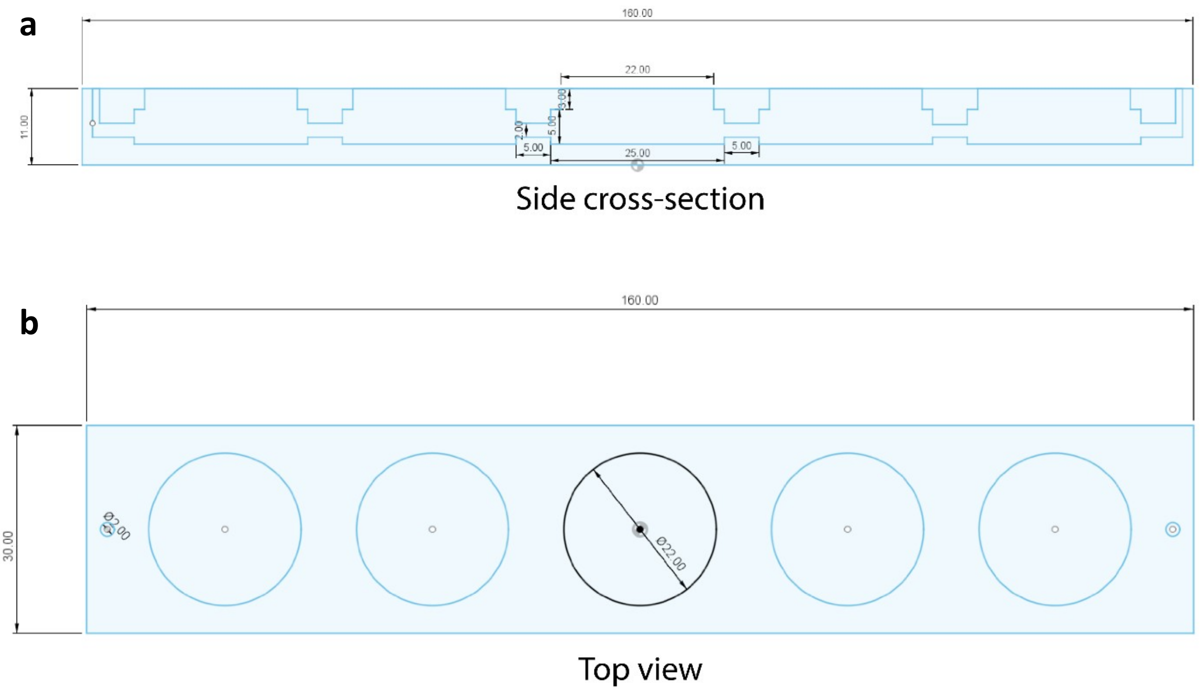

cascade is provided in Figure S1.

Figure S1. *Design sketch of the CSTR cascade: (a) side cross-sectional view and (b) top view.*

Nanoports (IDEX) were fixed to the inlet and outlet of the CSTR cascade, with the outlet connected to a syringe pump withdrawing at a flow rate of 200 μL/min for sample collection. The ultrasonic microreactor was operated under the optimized conditions: 15 W ultrasound power, 2 mg/mL Cyclosporin A, and 12 mg/mL PLGA. The residence time in the CSTR cascade was approximately 40 min. Nanospheres were then repeatedly washed with Milli-Q water and centrifuged to remove excess surfactant and free drug.

Nanoparticle characterization. The nanoparticles size distribution was determined by 3D cross-correlation dynamic light scattering (DLS) (LS Spectrometer, LS instruments, Switzerland). Particle morphology was assessed using scanning electron microscopy (SEM) (InspectF50A, FEI, Eindhoven, The Netherlands) and transmission electron microscopy (TEM) (FEI Company, Hillsboro, OR, USA). The detailed sample preparation and measurement conditions were described in our previous work [1]. For Brunauer–Emmett–Teller (BET) analysis, the CyA-PLGA NP suspension was freeze-dried for 3 days, followed by degassing at 25 °C for 12 hours. The adsorption isotherms were measured using N_2 at standard temperature and pressure (STP, 273 K and 101.325 kPa) with a surface area and pore size analyzer (Micromeritics ASAP 2020) to determine pore volume and pore size distribution.

Quantification of drug loading and encapsulation efficiency. Drug loading (DL) is defined as the percentage of drug content relative to the total mass of drug-loaded nanoparticles:

$$DL(\%) = \frac{encapsulated\ CyA}{total\ weight\ of\ nanospheres} * 100$$

Encapsulation efficiency (EE) is the ratio of encapsulated drug to the total amount of drug initially added:

$$EE(\%) = \frac{encapsulated\ CyA}{total\ CyA} * 100 \quad (2)$$

To quantify the encapsulated CyA, the washed nanospheres were dried in a vacuum chamber. A known mass of nanoparticles was dissolved in 1 mL of acetonitrile under stirring for 2 hours. Following this, 0.25 mL of methanol was added, and the mixture was stirred for 1 hour to precipitate the PLGA. The solution was centrifuged at 10,000 g for 30 min, and the supernatant was filtered using a 0.2 µm nylon syringe filter (Whatman) to remove the residual polymer.

The CyA concentration was measured using high-performance liquid chromatography (HPLC) (Shimadzu Prominence-i LC-2030C 3D Plus, HPLC-UV). A Zorbax Eclipse Plus reverse-phase C18 column (4.6 × 250 mm, 5 µm filler particle) was employed. The mobile phase consisted of a 90:10 (v/v) mixture of acetonitrile and Milli-Q water. The flow rate was set to 1 mL/min, with a column temperature of 40°C and using a detection wavelength of 210 nm. A calibration curve for a CyA solution in an acetonitrile-methanol mixture (4:1, v/v) over a concentration range of 1.56 - 200 µg/mL was prepared. Each condition was analyzed in triplicate to determine DL and EE values.

Quantification of organic solvent residual. The residual ethyl acetate in the nanosphere suspension after solvent evaporation was quantified using HPLC. To prepare the samples, 1 mL of acetonitrile was added to 0.5 mL of the nanoparticle suspension and stirred for 2 hours to fully dissolve the particles. Following this, 0.25 mL of methanol was added to precipitate the polymer and surfactant prior to HPLC analysis. The mixture was centrifuged at 10,000 g for 30 min, and the supernatant was filtered through a 0.2 µm nylon syringe filter to remove any remaining polymer.

HPLC analysis was performed using a Zorbax Eclipse Plus reverse-phase C18 column (4.6 × 250 mm, 5 µm filler particle). The mobile phase was a 10:90 (v/v) mixture of acetonitrile and Milli-Q water, with a flow rate of 1 mL/min. The column temperature was set to 40°C, with a detection wavelength of 204 nm. A calibration curve for ethyl acetate in water was established over a concentration range of 1.25 - 40 µL/mL. Each sample was analyzed in triplicate.

Additionally, residual ethyl acetate in each CSTR during continuous solvent evaporation was measured. A 0.25 mL sample was collected from each CSTR well, and the preparation and HPLC analysis followed the same procedure as described above, with the acetonitrile and methanol volumes halved to accommodate the smaller sample volume.

***In vitro* drug release study.** The *in vitro* release of encapsulated CyA from the nanospheres was studied using the dialysis bag diffusion method. Phosphate buffer saline (PBS, 0.01 M, pH 7.4) was used as the release medium. A known amount of the nanosphere suspension was dispersed in 2 mL of PBS and placed inside a dialysis bag (molecular weight cut-off ~ 12,400 Da). The dialysis bag was then submerged in a sealed glass bottle containing 18 mL of PBS, sufficient for establishing sink conditions (defined as the volume of medium at least three times that required to form a saturated drug solution [2,3]). The bottle was placed on a magnetic stirrer, maintaining a temperature of 37 ± 1°C with gentle stirring at 100 rpm. At predetermined time intervals, 0.5 mL samples were withdrawn and replaced with an equal volume of fresh PBS. The concentration of CyA in the collected samples was analyzed using HPLC to determine the amount of drug released. The cumulative CyA release from the nanospheres was calculated as:

$$cumulative\ release\ (\%) = \frac{M_t}{M_{total}} * 100 \quad (3)$$

where M_t is the amount of CyA released from the nanoparticles at time t, and M_{total} is the total amount of CyA encapsulated in the nanoparticles. The cumulative drug release percentage was

plotted over time. All release experiments were performed in triplicate, with the average values reported.

Statistical analysis. Statistical analyses were performed in GraphPad Prism 10. The effects of solvent evaporation temperature in the CSTR on nanoparticle synthesis were analyzed using a non-parametric unpaired t-test, assuming equal variance. *In vitro* drug release data were assessed through multiple comparisons using two-way ANOVA. Results with $P < 0.05$ were considered statistically significant.

SUPPLEMENTARY DATA SECTION

Solvent removal efficiency comparison: traditional batch removal v.s. thermal-controlled CSTR cascade

Table S1. *Comparison of solvent removal efficiency between batch processing in a glass vial and continuous processing in a CSTR cascade at room temperature (~23°C).*

Solvent removal setup	Emulsion volume [mL]	Surface area [mm^2]	Surface-to-volume ratio [mm^{-1}]	Evaporation time [min]	Residual ethyl acetate [µL/mL]
Batch vial	2.5	300	0.12	40	141.3 ± 9.1
CSTR cascade	8	1900	0.24	40	8.0 ± 1.2

Optimization of CyA-PLGA NP synthesis: batch solvent removal

Table S2 *Mean particle size, polydispersity index (PDI), drug loading (DL), and encapsulation efficiency (EE) of Cyclosporin A-loaded PLGA nanoparticles prepared by batch solvent evaporation. Data are presented as mean ± SD (n = 3).*

Power [W]	Cyclosporin A concentration [mg/mL]	PLGA concentration [mg/mL]	Mean particle size [nm]	PDI [-]	DL [%]	EE [%]
**10**	1	12	77.9 ± 2.1	0.13 ± 0.04	3.5 ± 0.1	43.8 ± 0.9
**15**	1	12	74.0 ± 2.7	0.16 ± 0.02	3.3 ± 0.2	41.4 ± 2.3
**20**	**1**	12	73.5 ± 1.6	0.11 ± 0.07	3.5 ± 0.1	43.0 ± 1.8
15	**2**	12	75.9 ± 1.5	0.10 ± 0.02	7.0 ± 1.2	45.1 ± 8.6
15	**3**	**12**	80.7 ± 1.5	0.08 ± 0.01	5.8 ± 0.5	24.4 ± 2.4
15	2	**24**	109.5 ± 0.3	0.11 ± 0.04	4.5 ± 0.1	56.5 ± 0.9
15	4	**48**	153.8 ± 2.0	0.11 ± 0.06	4.7 ± 0.1	59.1 ± 1.7

The objective was to identify the optimal synthesis conditions of the ultrasonic microreactor for producing nanospheres smaller than 100 nm, while maximizing the drug loading. The solvent evaporation step was carried out in batch using glass vials at room temperature (~23°C). Three key parameters - ultrasound power, the CyA to PLGA ratio, and PLGA concentration in the organic phase - were selected for an optimization based on our previous research[1]. Table S2 summarizes the results of particle size distribution, drug loading, and encapsulation efficiency.

First, the influence of ultrasound power was investigated, while maintaining CyA and PLGA concentrations at 1 mg/mL and 12 mg/mL, respectively. At 10 W, PLGA nanospheres with an average size of 77. 9 nm and a drug loading of 3.5% were produced. Increasing the power to 15 W reduced the mean particle size to 74.0 nm, with a comparable drug loading of 3.3%. However, further increasing the power to 20 W had no significant impact on either particle size or drug loading. These results indicate that 15 W of ultrasound power is sufficient for complete emulsification, producing fine droplets and small-sized nanospheres. Previous studies have shown that beyond a certain threshold, increasing ultrasound power can lead to more frequent droplet collisions and eventual coalescence[4,5]. Additionally, the larger number and size of cavitation bubbles at higher power can dampen the ultrasound effect, resulting in larger emulsion droplets[6]. This combination of droplet re-coalescence and ultrasound damping likely explains the lowered effectiveness in particle size reduction when increasing the power from 15 W to 20 W. Additionally, increasing the power to 20 W risks applying excessive energy, potentially leading to drug degradation. Thus, 15 W was selected as the optimal ultrasound power for further optimization of the remaining two parameters.

Next, the effect of the CyA to PLGA ratio was explored, with the ultrasound power set at 15 W. Increasing the CyA : PLGA ratio from 1 mg/mL : 12 mg/mL to 2 mg/mL : 12 mg/mL

significantly improved drug loading from 3.3% to 7.0%, with only a slight increase in mean particle size from 74.0 nm to 75.9 nm. However, further increasing the ratio to 3 mg/mL : 12 mg/mL led to a reduction of drug loading to 5.8%, while mean particle size increased further to 80.7 nm. The increase in the particle size is likely due to the higher viscosity of the organic phase at elevated CyA concentrations, which hinders emulsification. The results suggest that a CyA : PLGA ratio of 2 mg/mL : 12 mg/mL offers the optimal balance, yielding the highest drug loading, and this ratio was selected for the final parameter optimization.

Last, the effect of PLGA concentration was explored while maintaining the ultrasound power at 15 W and the CyA : PLGA ratio at 2 mg/mL : 12 mg/mL. PLGA concentrations of 12, 24, and 48 mg/mL were tested. Increasing the PLGA concentration from 12 to 48 mg/mL improved the encapsulation efficiency from 45.1% to 59.1%. However, the mean particle size increased substantially from 75.9 nm to 153.8 nm due to the higher viscosity of the organic phase at higher PLGA concentrations. Additionally, drug loading decreased from 7.0% to 4.7%. As a result, a PLGA concentration of 12 mg/mL and CyA concentration of 2 mg/mL were selected as the optimal condition to maintain the mean particle size below 100 nm while achieving both a high drug loading and encapsulation efficiency.

In conclusion, the optimal synthesis conditions for the ultrasonic microreactor were determined to be ultrasound power of 15 W, CyA concentration of 2 mg/mL, and PLGA concentration of 12 mg/mL. Notably, all PLGA nanospheres synthesized in this study exhibited a PDI below 0.2, meeting the monodispersity standards required for biomedical applications.

Blank PLGA nanospheres

Table S3. *Mean particle size and polydispersity index (PDI) of blank PLGA nanospheres prepared by batch solvent evaporation. Data are presented as mean ± SD (n = 3).*

Power<br>[W]	PLGA concentration<br>[mg/mL]	Mean particle size<br>[nm]	PDI<br>[-]
10	12	83.58 ± 4.02	0.10 ± 0.02
15	12	78.06 ± 1.90	0.21 ± 0.03
20	12	80.59 ± 1.25	0.16 ± 0.07

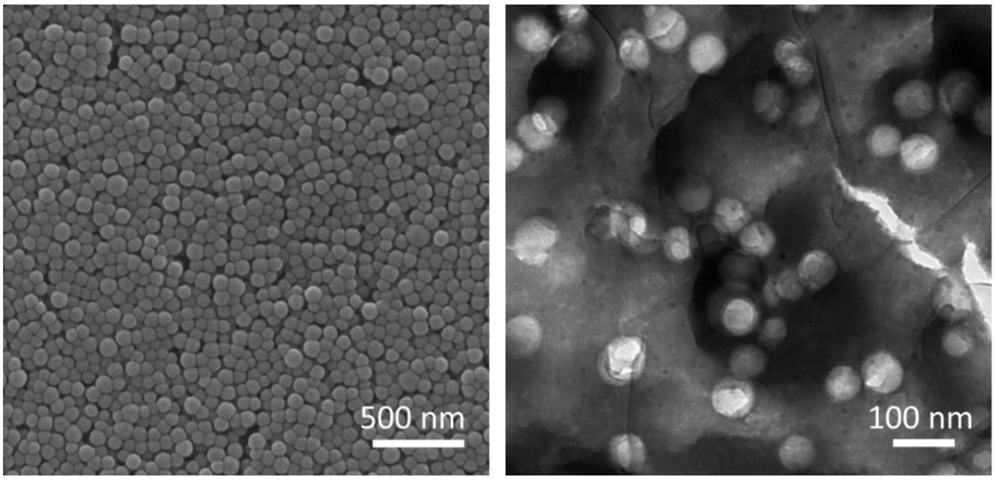

Figure S2. *SEM (left) and TEM (right) images of blank PLGA nanospheres prepared by batch solvent removal (ultrasound power: 15W).*

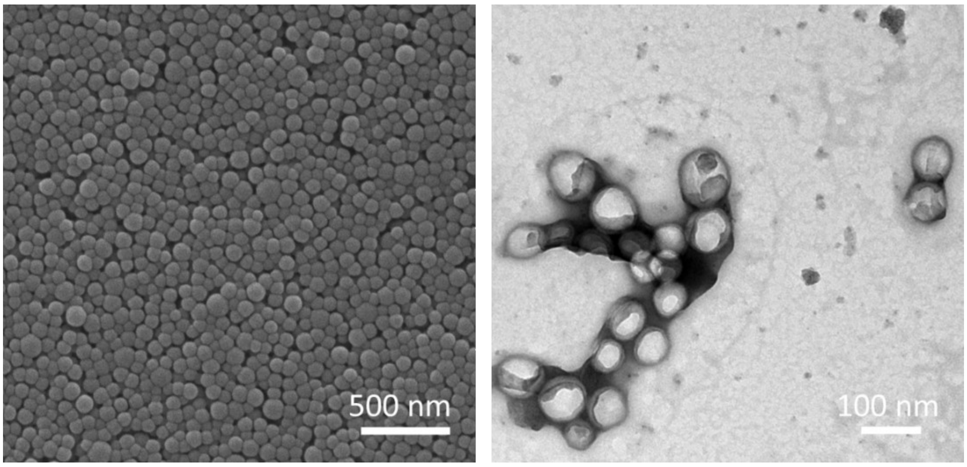

CyA-PLGA NPs prepared by batch solvent removal: SEM & TEM

Figure S3. *SEM (left) and TEM (right) images of CyA-PLGA NPs prepared by batch solvent removal. Conditions: ultrasound power of 15W, CyA concentration of 2 mg/mL, and PLGA concentration of 12 mg/mL.*

CyA degradation in the ultrasonic microreactor

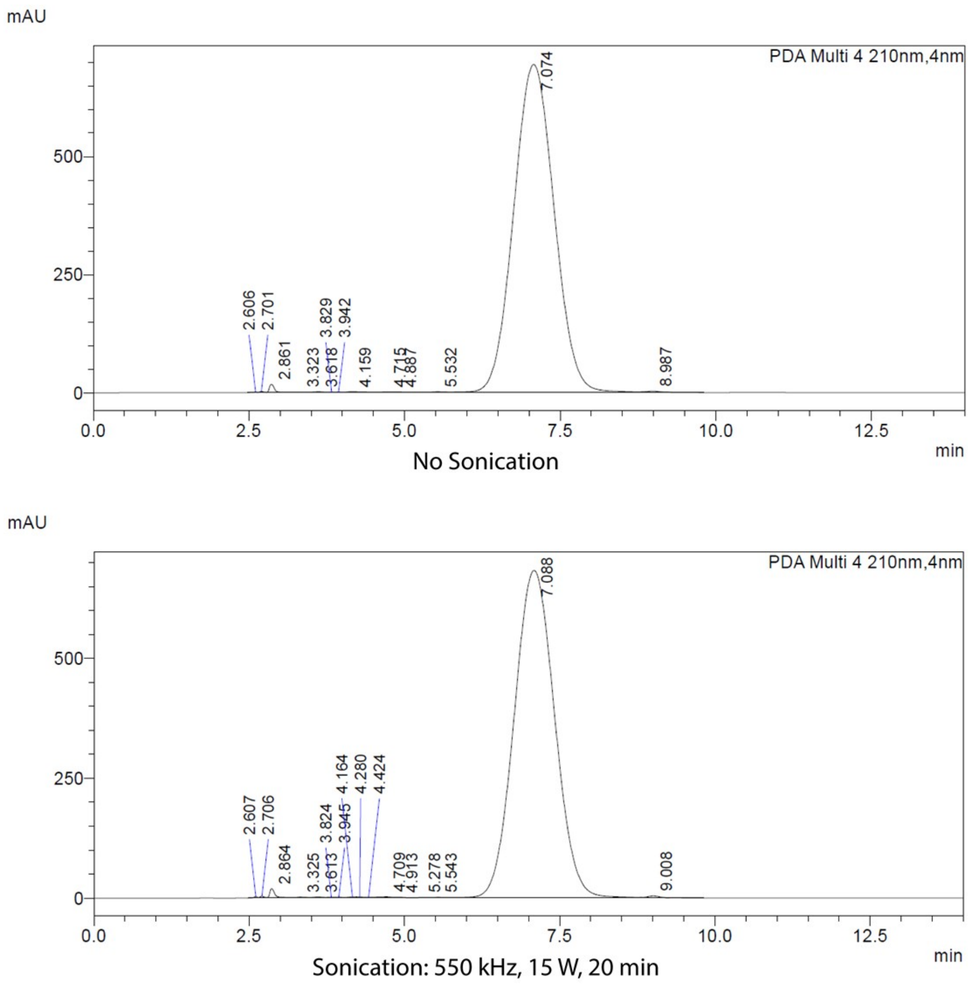

Figure S4. *HPLC chromatogram of CyA at a detector wavelength of 210 nm: (a) before sonication and (b) after sonication.*

To test the degradation of CyA in the ultrasonic microreactor, a 1 mg/mL solution of CyA in acetonitrile was prepared. This solution was pumped through the ultrasonic microreactor at a flow rate of 50 μL/min, with a residence time of 20 min (5 times the residence time during nanoparticle synthesis). For the sonicated sample, ultrasound was activated at a frequency of 550 kHz and a power of 15 W. Samples were collected after 40 min, with approximately 1.5 mL of each sample in a closed HPLC vial. Similarly, 1.5 mL sample was collected for non-sonicated condition. From both the sonicated and non-sonicated samples, 1 mL was taken and mixed with 0.25 mL of methanol. The mixtures were stirred for 30 minutes and filtered through a 0.2 μm syringe filter before HPLC analysis.

HPLC measurements for the non-sonicated sample were repeated five times, yielding an average peak area of 31,418,235 (SD = 112,338). The peak area for the sonicated sample was 30,970,475, indicating that CyA degradation was less than 1.5%.

HPLC protocol: encapsulation efficiency and drug loading

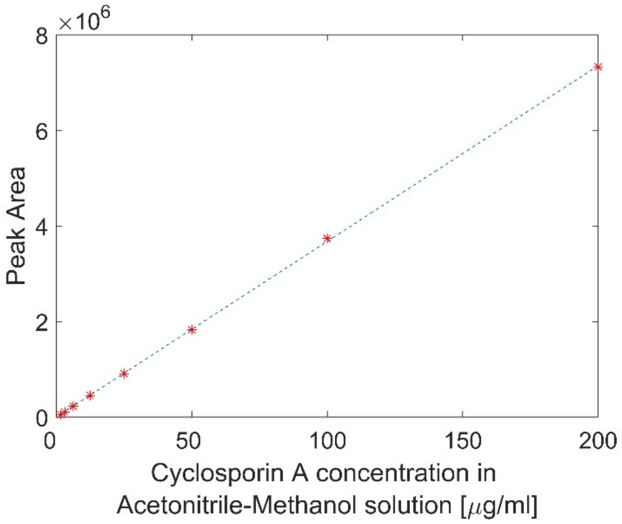

Figure S5. *HPLC calibration curve for CyA concentration in acetonitrile: methanol = 4:1 (v/v) solution, obtained in the concentration range of 1.56-200 µg/ml. Peak Area = 36774*CyA concentration (R^2 = 0.9999).*

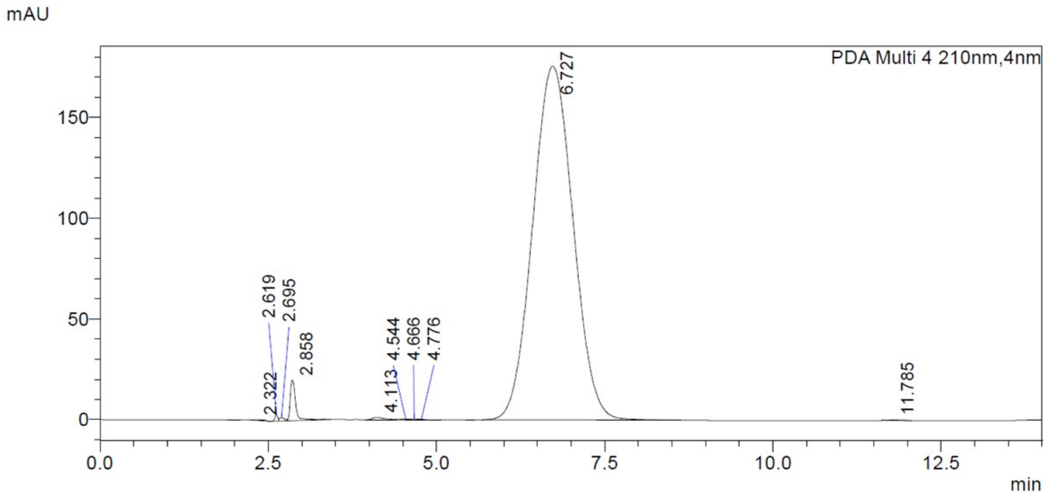

Figure S6. *HPLC chromatogram of CyA in acetonitrile: methanol = 4:1 (v/v) solution at a detector wavelength of 210 nm. The CyA concentration is 200 µg/mL and the retention time is 6.727 min.*

HPLC protocol: residual ethyl acetate in CyA PLGA NP suspension

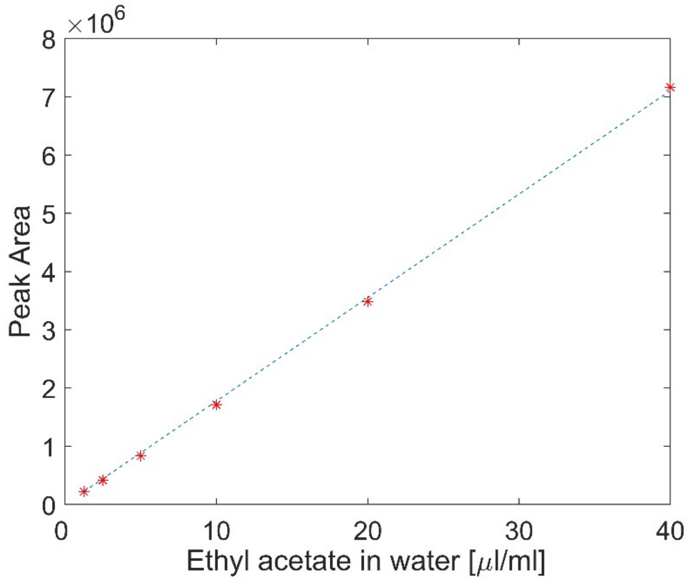

Figure S7. *HPLC calibration curve of ethyl acetate concentration vs. peak area in in Milli-Q water obtained in the concentration range of 1.25-40 µl/ml. Peak Area = 1.7763*10*^{6}**ethyl acetate concentration (*R^2 *= 0.9996).*

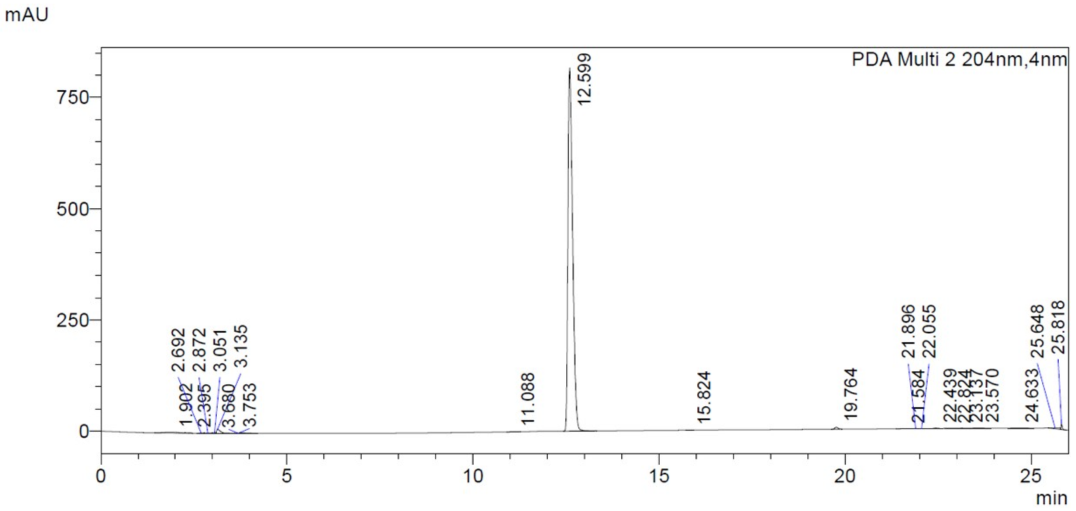

Figure S8. *HPLC chromatogram of ethyl acetate in Milli-Q water (40 µL/mL) at a detector wavelength of 204 nm. The retention time is 12.599 min.*

CyA degradation during *in vitro* drug release study

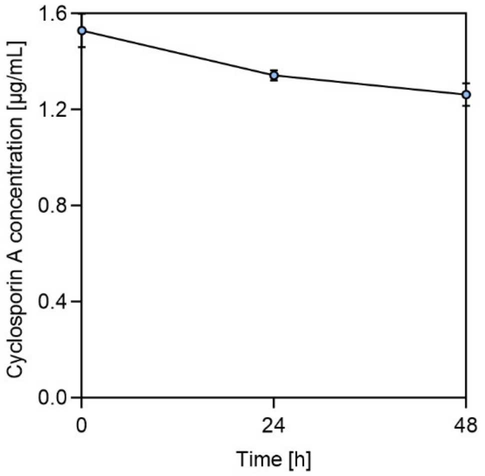

Figure S9. *CyA concentration during degradation test under the same condition as the in vitro drug release study (PBS 0.01M, 37 °C). Data are plotted as mean ± SD (n = 3).*

To evaluate the degradation of CyA during the drug release study, a solution of CyA in PBS (0.01 M, pH 7.4) was prepared at a concentration of 1.5–2 µg/mL, mirroring the peak concentration observed in the release experiment (approximately 2 µg/mL). A 20 mL of this solution was placed in the same setup that was used in the release study, with the temperature maintained at 37 °C and gentle magnetic stirring. At 24 h and 48 h, 0.5 mL samples were withdrawn, mixed with 0.5 mL of acetonitrile, and subsequently analyzed by HPLC to determine drug concentration. The results demonstrated a gradual degradation of CyA over time, consistent with degradation observed during the drug release study.

Temperature effects on nanosphere morphology

Figure S10 presents SEM and TEM images of CyA-PLGA NPs synthesized via continuous solvent removal in the CSTR cascade at different temperatures. The nanospheres display a smooth, spherical morphology with a uniform size distribution ranging from 70 to 85 nm, confirming the DLS measurements. No significant differences in morphology are observed between nanoparticles synthesized at ~23°C and 30°C, indicating that the soft thermal treatment does not compromise particle integrity. Moreover, the ability to achieve controlled polymer crystallization and solvent evaporation in a continuous process marks a significant advancement, as it preserves the structure of the particles and enhances drug loading efficiency. However, at 40°C and 45°C, the number density of particle decreases significantly, with some particles exhibiting an irregular shape. This difference is due to the loss of PLGA material during solvent evaporation, caused by the transition of PLGA into its rubbery state above T_g', which also correlates with the observed decrease in drug loading.

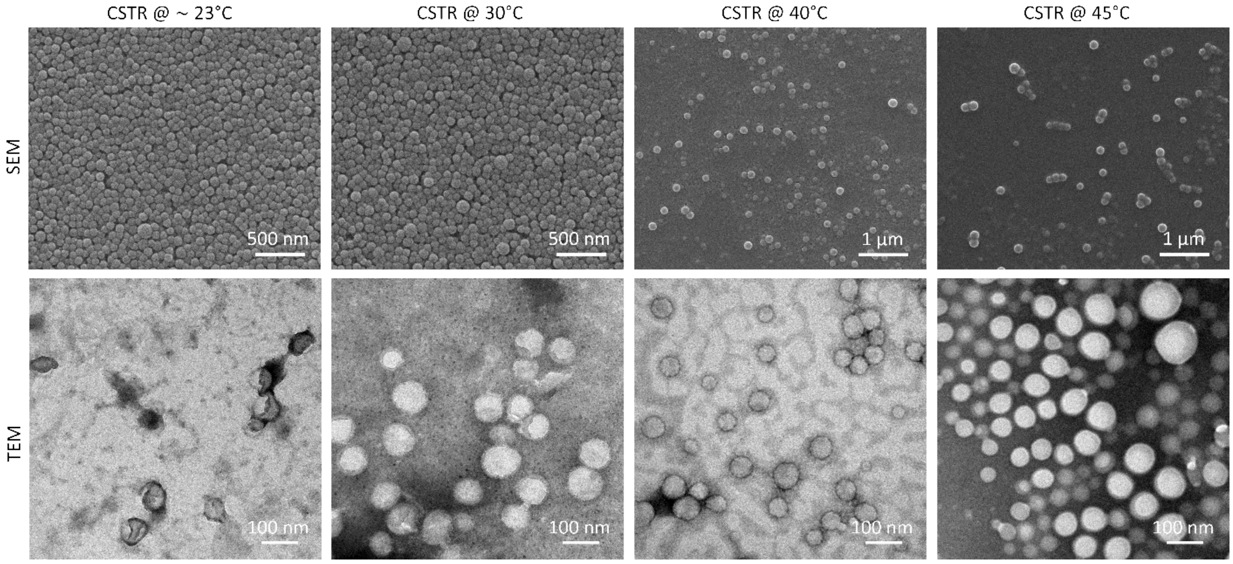

Figure S10. *Representative SEM and TEM images of CyA-PLGA NPs synthesized by thermal-controlled CSTR cascade at room temperature, 30°C, 40°C, and 45°C (CSTR @ ~23°C, CSTR @ 30°C, CSTR @ 40°C, and CSTR @ 45°C).*

Numerical data of Figure 1c and Figure 2e-f

Table S4. *Mean particle size, polydispersity index (PDI), drug loading (DL), and encapsulation efficiency (EE) of CyA-PLGA NPs prepared via thermal-controlled CSTR cascade at various temperatures, and by conventional batch method at 35 °C for 40 min and 4 h. Data are presented as mean ± SD (n = 3).*

Solvent removal conditions	Mean particle size [nm]	PDI [-]	DL [%]	EE [%]
CSTR @ ~23°C	82.6 ± 1.3	0.19 ± 0.02	4.2 ± 1.3	26.5 ± 8.5
CSTR @ 30°C	82.3 ± 1.6	0.08 ± 0.05	5.8 ± 0.4	38.4 ± 3.1
CSTR @ 35°C	79.4 ± 8.1	0.15 ± 0.03	7.6 ± 0.4	49.0 ± 2.5
CSTR @ 40°C	75.1 ± 0.8	0.19 ± 0.00	3.6 ± 0.7	22.7 ± 4.6
CSTR @ 45°C	76.7 ± 0.6	0.19 ± 0.02	3.9 ± 0.3	24.4 ± 2.2
Batch @ 35°C (40 min)	78.3 ± 2.9	0.19 ± 0.03	0.8 ± 0.1	5.0 ± 0.6
Batch @ 35°C (4 hours)	78.5 ± 1.1	0.13 ± 0.02	2.5 ± 0.9	15.3 ± 5.7

Numerical data of Figure 3c-d

Table S5. *Ethyl acetate residual content in the CyA PLGA NP suspension prepared by continuous solvent removal in a CSTR cascade at different temperatures and batch solvent removal at 35°C for 40 min and 4 h. Data are presented as mean ± SD (n = 3).*

**Solvent removal conditions**	**Residual ethyl acetate [µL/mL]**
CSTR @ ~23°C	8.0 ± 1.2
CSTR @ 30°C	2.3 ± 0.3
CSTR @ 35°C	0.9 ± 0.4
CSTR @ 40°C	1.5 ± 0.9
CSTR @ 45°C	2.4 ± 0.1
Batch @ 35°C (40 min)	91.9 ± 1.6
Batch @ 35°C (4 h)	1.0 ± 0.4

**CSTR well**	**Ethyl acetate concentration [µL/mL]**				
	CSTR @ ~23°C	CSTR @ 30°C	CSTR @ 35°C	CSTR @ 40°C	CSTR @ 45°C
1	122.41	66.31	62.06	54.03	48.49
2	63.62	33.61	22.44	18.31	8.71
3	34.08	11.40	6.91	5.36	3.46
4	17.16	4.52	2.30	4.10	1.36
5	8.87	1.92	0.84	2.67	0.75

Table S6. *Residual ethyl acetate levels in each CSTR well throughout the evaporation process at different temperatures.*

Numerical data of Figure 4b-c

Table S7. *In vitro drug release profiles of CyA-PLGA NPs prepared by thermal-controlled CSTR cascade at 35 °C (CSTR @ 35 °C), compared with (a) batch solvent removal at room temperature (Batch @ ~23 °C), and (b) batch solvent evaporation at 35 °C (Batch @ 35 °C). Continuous solvent removal was conducted for 40 min, while both batch processes were extended to 4 h to ensure sufficient drug encapsulation for quantifiable release measurements. Data are plotted as mean ± SD (n = 3).*

**Time [h]**	**Cumulative release [%]**		
	**CSTR @ 35°C (40 min)**	**Batch @ ~23°C (4 h)**	**Batch @ 35°C (4 h)**
3	40.9 ± 0.5	31.3 ± 3.6	40.5 ± 8.3
6	54.5 ± 4.5	45.0 ± 3.2	57.6 ± 6.2
9	60.8 ± 1.4	53.7 ± 9.8	86.2 ± 10.4
24	71.2 ± 2.3	80.0 ± 1.5	68.3 ± 13.3
48	75.9 ± 4.8	88.9 ± 3.8	82.1 ± 12.5
72	72.3 ± 2.6	92.5 ± 3.1	96.3 ± 3.7
96	69.2 ± 4.4	91.3 ± 0.5	90.6 ± 8.9
120	65.9 ± 3.6	84.7 ± 1.1	72.5 ± 8.4

References:

(1) Udepurkar, A. P.; Mampaey, L.; Clasen, C.; Sebastián Cabeza, V.; Kuhn, S. Microfluidic Synthesis of PLGA Nanoparticles Enabled by an Ultrasonic Microreactor. *React. Chem. Eng.* **2024**, *9*, 2208-2217.

(2) The United States Pharmacopeia: The National Formulary (USP37/NF32); The United States Pharmacopeial Convention, Inc.: Rockville, MD, 2014.

(3) A. Abouelmagd, S.; Sun, B.; C. Chang, A.; Jin Ku, Y.; Yeo, Y. Release Kinetics Study of Poorly Water-Soluble Drugs from Nanoparticles: Are We Doing It Right? *Mol. Pharm.* **2015**, *12* (3), 997–1003.

(4) Gaikwad, S. G.; Pandit, A. B. Ultrasound Emulsification: Effect of Ultrasonic and Physicochemical Properties on Dispersed Phase Volume and Droplet Size. *Ultrason. Sonochem.* **2008**, *15* (4), 554–563.

(5) Canselier, J. P.; Delmas, H.; Wilhelm, A. M.; Abismaïl, B. Ultrasound Emulsification - An Overview. *J. Dispers. Sci. Technol.* **2002**, *23* (1–3), 333–349.

(6) Brotchie, A.; Grieser, F.; Ashokkumar, M. Effect of Power and Frequency on Bubble-Size Distributions in Acoustic Cavitation. *Phys. Rev. Lett.* **2009**, *102*, 084302.
